# Modified Ludloff’s medial approach for resection of heterotopic ossification of the hip following severe SARS-CoV-2 infection: a case report

**DOI:** 10.1093/jhps/hnad048

**Published:** 2024-01-04

**Authors:** Ricardo Ramón, Esteban Holguín, Manuel Ribas, Nihad Al Hussin, Marco Ezechieli

**Affiliations:** Clinic for Orthopedic Surgery, Trauma and Sports Medicine, St. Vincenz Hospital Location Salzkotten, Salzkotten, 33154, Germany; Orthopedics Surgery, Trauma, Sports Medicine and Orthopedic Oncology, QRA, Quito 170184, Ecuador; Hip Surgery ICATME, Dexeus University Hospital, Barcelona 08028, Spain; Clinic for Orthopedic Surgery, Trauma and Sports Medicine, St. Vincenz Hospital Location Salzkotten, Salzkotten, 33154, Germany; Clinic for Orthopedic Surgery, Trauma and Sports Medicine, St. Vincenz Hospital Location Salzkotten, Salzkotten, 33154, Germany

## Abstract

The coronavirus disease 2019 pandemic has significantly affected people worldwide. Herein, we present a case of massive heterotopic ossification (HO) of the right hip following severe SARS-CoV-2 infection. The exact origin of HO development is still unknown, but a critical illness, chronic immobilization and hypoxia are important risk factors. Considering the location and size of the HOs in this case, modified Ludloff’s medial approach of the hip was used. This approach allows for good exposure and access to the medial and inferior part of the hip joint and the successful extirpation of the pathologic tissue.

## INTRODUCTION

### Heterotopic ossification

Heterotopic ossification (HO) was first described by Riedel in 1883 as the formation of mature, lamellar bone in soft tissues without ossification properties [1–3]. The exact etiology of HO has not been specifically described; it could be multifactorial and associated with many risk factors [3]. It is often related to trauma or prior surgeries, but it has also been associated with congenital disorders, metabolic disorders and brain injury and infrequently reported after immobilization due to critical illness [4–7].

The most commonly affected joints are the hip in the abductor compartment, elbow and shoulder [2, 3]. HO usually appears 8–10 weeks after the metabolic disturbance or provoked injury [1, 8]. Conventional radiographic imaging helps in the diagnosis and follow-up of HO, but computed tomography (CT) with 3D reconstruction is the gold standard for accurate location and surgical planning [4, 5].

Experiments in the 1950s showed good sensitivity of osteogenic progenitors to radiation therapy, since then low-dose radiation is used as a prophylactic treatment to decrease the chance of HO formation [2, 9]. The most effective treatment is preoperative radiotherapy soon before the procedure, maximum 24 h preoperatively or 72 h postoperatively with a prescribed dose from 7 to 8 Gy [2, 9]. A single-dose radiotherapy is a safe and reliable method with a low rate of HO relapse [10].

In December 2019, a novel virus was identified, which caused pneumonia and severe acute respiratory syndrome (SARS) in many patients, subsequently designated as severe acute respiratory syndrom coronavirus 2(SARS-CoV-2). The spread of SARS-CoV-2 led to a global crisis, and coronavirus disease 2019 (COVID-19) was eventually declared a pandemic [11, 12]. Since then, cases of HO after severe COVID-19 infections which required mechanical ventilation were reported.

After a literature review: Aziz *et al.*, Meyer *et al.*, Van Ochter *et al.* and Brance *et al.* reported different cases. The symptoms and diagnosis of HO started only after extubation of the patients or were discovered as an incidental finding [7, 13–15]. In all these studies, possible treatments and preventions are discussed, but the different surgical techniques for the HO resection, especially in the hip, are not identified or mentioned.

### Medial hip approach

Ludloff first described the ‘medial’ or ‘trans adductor approach’ in 1908 [16, 17]. The original approach describes the superficial intermuscular plane between the sartorius and the adductor longus and the deeper interval between the pectineus and iliopsoas [17, 18]. In 1973, it was modified by Ferguson to a more medial approach, where the superficial interval was the same, but the deep interval was between the adductor brevis and adductor magnus [18, 19]. Some years later, Chiron described another more anterior located interval allowing a more minimally invasive access to the joint. All these approaches provide a proper view of the inferior joint region. Nevertheless, the risk of an obturator nerve injury is still present [17, 19].

## CASE REPORT

The patient was informed that data concerning the case would be submitted for publication, and he agreed; written consent was obtained. One week after a positive COVID-19 diagnosis, a 53-year-old male patient with no remarkable past medical history was admitted to the hospital with a SARS-CoV-2 diagnosis. Due to increased dyspnea and worsening hypoxia, he was quickly transferred to the intensive care unit (ICU). During the ICU stay, the patient developed septic shock and had a stroke. During his month at the ICU, he received long-term ventilation, extracorporeal membrane oxygenation therapy, tracheotomy and prolonged weaning. Finally, he was discharged to a neurological early rehabilitation center.

**Fig. 1.**
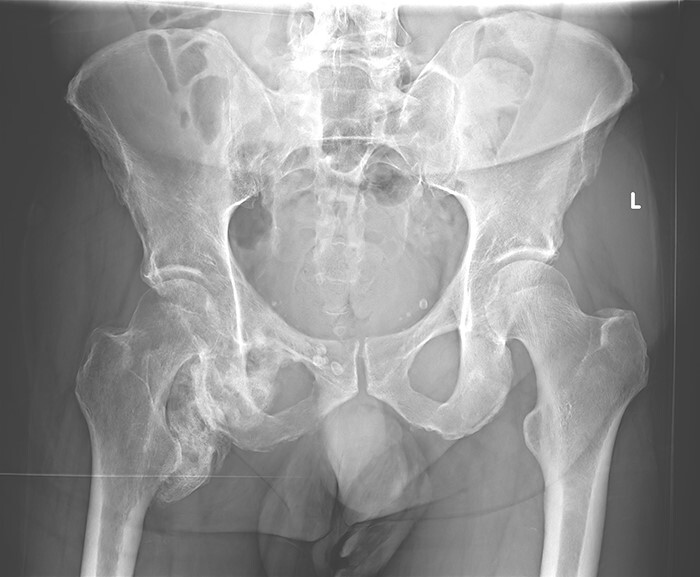
Pelvic overview, HO around the right hip. Patient at 6 months after therapy in the ICU.

**Fig. 2.**
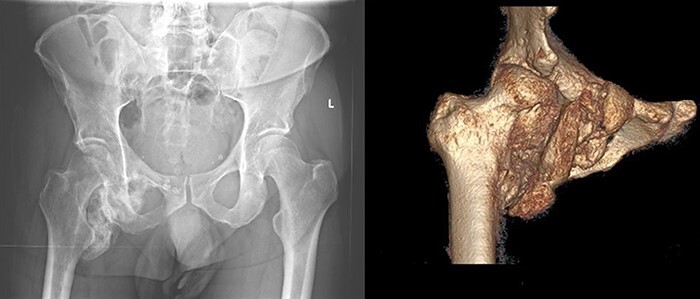
Pelvic overview radiograph and 3D reconstruction, 10 months after ICU therapy. Peripheral density of calcification in the medial/inferior area from the right hip, corresponding to HO.

Six months after his initial treatment, he presented to our orthopedic outpatient clinic, describing pain and mobility limitation of his right hip. He showed right hemiparesis after suffering a left-brain stroke as a thromboembolic complication from his SARS-CoV-2 infection. In the physical examination, he had swelling in the upper medial thigh, marked limited range of motion and pain. The hip radiographs (Fig. 1) showed a significant formation of HO in the right hip. The patient was still in neurological recovery; hence, the direct operative treatment was postponed. However, as per the published literature, the earliest operative treatment should be offered between 9 and 12 months after the emergence of HO [3]. After 5 months, his clinical symptoms worsened. The new radiographs showed an increase in the HO. Complementary imaging with CT scan and 3D reconstruction was carried out (Fig. 2).

### Surgical technique

After carefully analyzing the localization of the HO, it was decided to access it through a medial hip approach. All variants of this approach were discussed and analyzed, and finally after considering all risks and benefits, it was decided to take modified Ludloff’s approach or Ferguson. Based on the 3D reconstruction, the HO is also directed toward the posterior part of the hip and the adductor magnus muscle. With the Chiron modification, it would not have been a sufficient certainty that the exposure of the HO in this specific case was going to be adequate since the preparation of the deep muscle plane is more anterior. During the surgery, the exposure and protection of the obturator nerve were always considered. In the morning, prior to the intervention, a radiation therapy of the right hip was performed; no further radiation therapy was given.

**Fig. 3.**
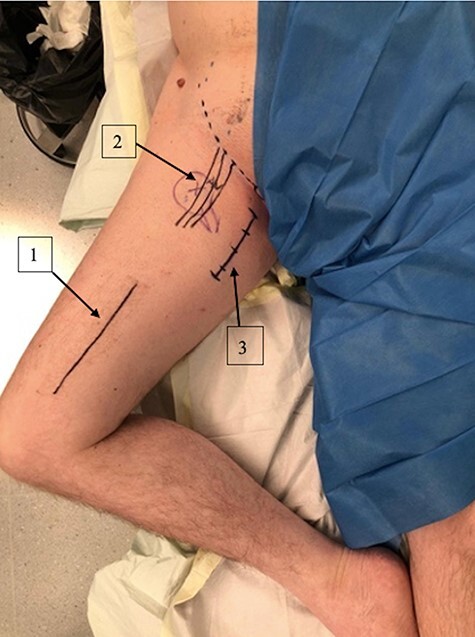
Patient in the ‘frog leg’ position with the approach landmarks. 1 = femur direction; 2 = from inside to outside: vein, artery nerve; 3 = medial hip approach.

**Fig. 4.**
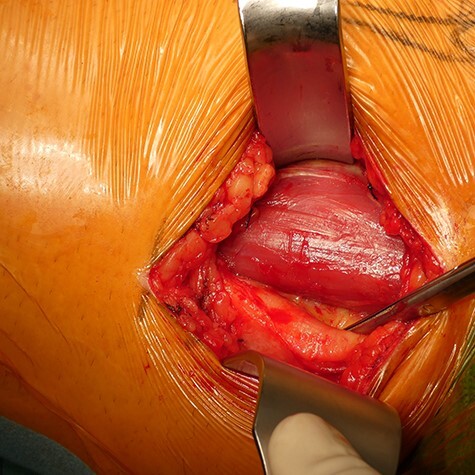
After draping and skin incision. An approximately 6- to 8-cm-long incision aiming toward the medial femoral condyle and following the adductor longus muscle was performed. Plane-by- plane preparation.

The patient was positioned supine under general anesthesia. The leg was placed in a ‘frog leg’ position (flexion, external rotation and abduction). Under sonographic guidance, identification and marking of femoral vessels were performed. The landmarks for the approach were the tuberculum pubicum and the tendon of the musculus adductor longus (Fig. 3). A 6- to 8-cm incision toward the medial femoral condyle and following the adductor longus muscle was performed. After subcutaneous preparation, the aponeurosis was incised longitudinally, and the superficial intermuscular plane was identified between the gracilis (medial) and the adductor longus (lateral) (Fig. 4). After retracting these muscles, the deep plane was identified between the adductor brevis and adductor magnus muscles, performing modified Ludloff’s approach. It is important to protect the posterior division of the obturator nerve.

**Fig. 5.**
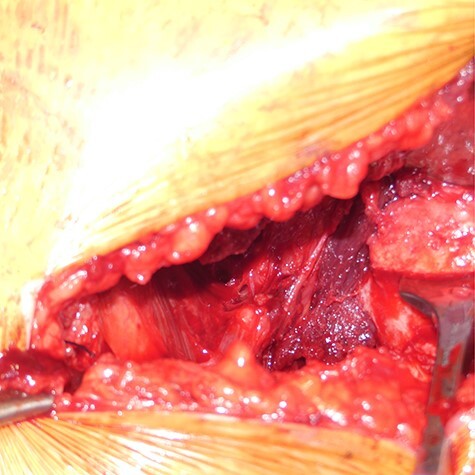
Modified Ludloff’s medial approach. Deep HO tissue around the right hip after adequate preparation.

**Fig. 6.**
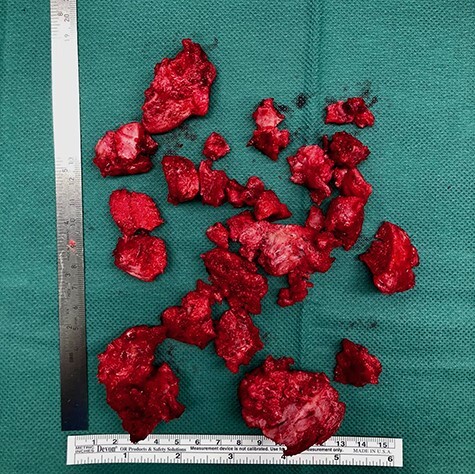
Approximately 18 × 14 cm HO tissue removed from around the right hip.

**Fig. 7.**
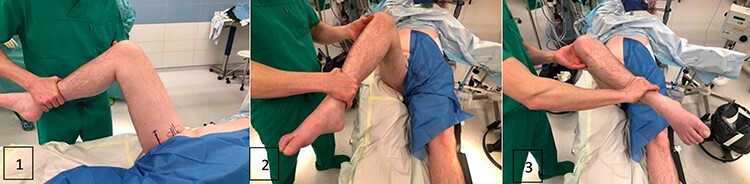
Preoperative limitation from the hip (1) hip flexion, (2) internal rotation and (3) external rotation.

**Fig. 8.**
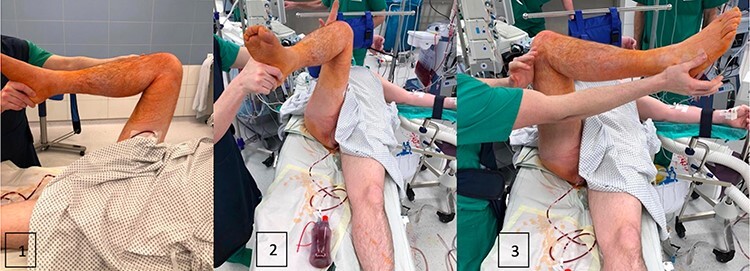
Postoperative mobility from the hip: (1) hip flexion, (2) internal rotation and (3) external rotation.

Retractors were placed on the superior and inferior aspects of the femoral neck, and the HO was carefully exposed; it started in the transition between the iliopsoas tendon and muscle. The HO extended distally from the lesser trochanter, proximally reaching the area of the ramus ossis pubis, laterally reaching the anterior aspect of the hip capsule, medially reaching the border of the adductor brevis muscle and on the bottom, reaching the obturator externus muscle (Fig. 5). After delimiting the borders of the malformation and trying to dissect and preserve all possible structures and soft tissue, an exhaustive step-by-step removal was carried out, and a capsulotomy was not required. Considering the size, the removal from HO was carried out in parts (Fig. 6). The wound was then flushed, a deep drain was placed and a layer-by-layer closure of the approach was performed. A hip range of motion preoperatively and postoperatively was performed (Figs 7 and 8).

**Fig. 9.**
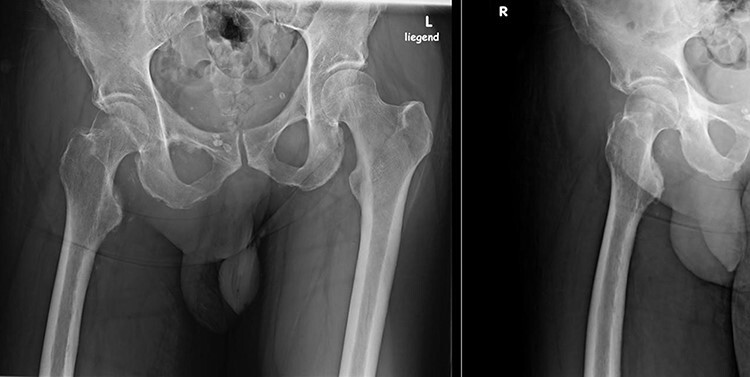
Postoperative pelvic overview radiography and Lauenstein view.

For further ossification prophylaxis, the patient received non-steroidal anti-inflammatory drugs (NSAIDs), in this case, etoricoxib, postoperatively for 3 weeks. In each case, the risks and benefits of administering NSAIDs must be evaluated. Mobilization was carried out with full weight bearing and a continuous passive motion (CPM) of the hip from the first postoperative day. On Day 7, after a postoperative radiography was done (Fig. 9), the patient was discharged.

**Fig. 10.**
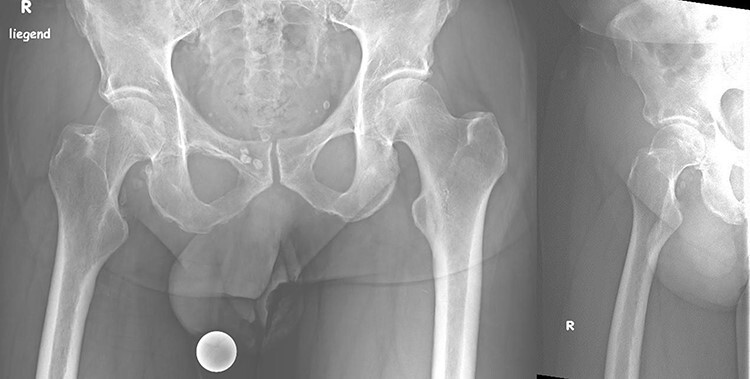
One-year postoperative radiograph: pelvic overview and Lauenstein view.

At a 1-year postoperative follow-up, the patient reported improved mobility and less pain than he had previously. The patient is primarily wheelchair-dependent due to his neurological sequelae, but a four-point gait is possible. Furthermore, in addition to the positive clinical outcome, the radiographs showed significant improvement when considering residual HO and absence of new HO formation (Fig. 10).

## DISCUSSION

Modified Ludloff’s approach was used in our patient because of its relative safety and direct access for the treatment of lesions located in the caudal and medial part of the hip joint. It is also a minimally invasive approach, which reduces the risk of neurovascular complications. Therefore, this approach has also been used for fracture management of the femoral head and hip arthroplasty [17–19]. Some advantages of this approach include direct access to femoral head, better exposure of the postero-medial area of the hip joint, preservation of the abductor compartment, limited blood loss and a relatively small scar [17–19].

The cytokine storm associated with COVID-19, persistent immobilization, post-stroke hemiplegia and hypoxia acted as trigger factors for HO formation in this patient [7, 20]. Furthermore, mechanical ventilation caused a homeostatic disbalance, causing modifications in the pH of the body. This imbalance associated with systemic alkalosis found in patients with acute respiratory distress syndrome triggers callus deposition, resulting in an increased HO development [21].

Brooker classification describes the HO in the hip, but this does not provide the specific location and does not guide a treatment or surgical hip approach. DeBraun et al. [4] suggested a new classification, which includes three categories based on the anatomic locations of the HO: anteriorly (Type 1), posteriorly (Type 2) and medially based (Type 3) [4]. For Type 1, the best approach is from the anterior side; for Type 2, through the posterior side and for Type 3, through the medial side [4].

The use of CPM after surgery improves the range of movement. Furthermore, etoricoxib was utilized due to its once-daily administration, well-established safety and efficacy in preventing HO; utilizing NSAIDs decreases the incidence of recurrent ossification [3, 22]. The risk of recurrence in these cases is much higher; therefore, preoperative radiation therapy and early physiotherapy are crucial. Planning prior to the surgery was important because a multidisciplinary team was necessary.

## CONCLUSION

Modified Ludloff’s approach is a very good and safe option for the treatment and removal of HO located in the medial compartment of the hip. This is a minimally invasive and muscle-preserving approach. A sonographic identification of the femoral vessels should be performed.

Considering the characteristics of the patient and the location and magnitude of the HO, the best approach for each individual patient should be considered. The diagnosis and awareness of HO formation are important, especially in patients with prolonged ventilation or critical care therapy and immobilization. The SARS-CoV-2 infection could be a new risk factor for HO formation; further research is needed to clarify any relation.

In the management of HO, prevention is key to avoid invasive treatment and surgeries. A complete surgical therapy for hip HO should include the following:

- Surgical planning and management by a multidisciplinary team.
- Preoperative imaging including 3D CT scan.
- Preoperative ossification-prophylactic irradiation directly prior to the procedure.
- A minimally invasive approach considering the location of HO.
- Postoperative use of CPM, physiotherapy and NSAID therapy is necessary and crucial for a successful surgery.

## Data Availability

The data underlying this article will be shared on reasonable request to the corresponding author.
